# Optical properties of as-grown and annealed InAs quantum dots on InGaAs cross-hatch patterns

**DOI:** 10.1186/1556-276X-6-496

**Published:** 2011-08-17

**Authors:** Chalermchai Himwas, Somsak Panyakeow, Songphol Kanjanachuchai

**Affiliations:** 1 Semiconductor Device Research Laboratory (Nanotec Center of Excellence), Department of Electrical Engineering, Faculty of Engineering, Chulalongkorn University, Bangkok 10330, Thailand

**Keywords:** quantum dots, cross-hatch patterns, photoluminescence, annealing, InAs, InGaAs.

## Abstract

InAs quantum dots (QDs) grown on InGaAs cross-hatch pattern (CHP) by molecular beam epitaxy are characterized by photoluminescence (PL) at 20 K. In contrast to QDs grown on flat GaAs substrates, those grown on CHPs exhibit rich optical features which comprise as many as five ground-state emissions from [1-10]- and [110]-aligned QDs, two wetting layers (WLs), and the CHP. When subject to *in situ *annealing at 700°C, the PL signals rapidly degrades due to the deterioration of the CHP which sets the upper limit of overgrowth temperature. *Ex situ *hydrogen annealing at a much lower temperature of 350°C, however, results in an overall PL intensity increase with a significant narrowing and a small blueshift of the high-energy WL emission due to hydrogen bonding which neutralizes defects and relieves associated strains.

## Introduction

Self-assembled InGaAs quantum dots (QDs) have been intensively investigated during the last decade due to their high crystalline quality [1]. InGaAs QDs conventionally grown on on-axis (100)-GaAs substrates are optically active and typically emit in the 1.0 to 1.3 eV range [2]. Those grown unconventionally - on high-index substrates [3], pre-patterned layers [4], or cross-hatch patterns [5-9] - exhibit similar optical characteristics with a possibility to obtain lateral QD alignment, further expanding the range of optoelectronic applications which includes lasers [10] and detectors [11]. These QDs are usually embedded in a junction/mirror structure and have to be overgrown by GaAs or AlGaAs. The active (QD) and overgrown layers, however, have different growth temperature requirements: QDs growth temperature is low (approximately 470°C to 520°C) to prevent In desorption, but subsequent overlayer growth temperature is high (580°C and above), especially if the layer contains slow-diffusing species such as Al. The fundamental difference in growth temperature requirements and its inevitability lead to extensive investigation of the properties of InGaAs QDs annealed *in situ *[12-15] and *ex situ *[15-21]. In terms of luminescence, it is well established that conventional InGaAs QDs that underwent annealing would: (1) exhibit a blueshift in their ground-state emission, (2) have narrower linewidth, and, in some cases, (3) emit at an increased intensity due to interdiffusion and intermixing of cations and the reduction in non-radiative recombination centers in the surrounding matrix [13-20]. Annealing studies of unconventional InGaAs QDs such as those grown on InGaAs metamorphic or cross-hatch patterns (CHPs), however, have received much less attention, partly because of the perceived inferiority due to the presence of misfit dislocations (MDs) at the InGaAs/GaAs heterointerface [22] and partly because the full explanation of the rich optical features of these types of QDs is still lacking.

In this paper, InAs QDs on InGaAs CHPs, controlled InAs QDs, and controlled InGaAs CHPs are grown by molecular beam epitaxy (MBE) and subject to high-temperature *in situ *and low-temperature *ex situ *annealing. The optical properties of the samples - as-grown and annealed - as characterized by photoluminescence (PL) show that QDs on CHP have rich optical features and that high-temperature *in situ *annealing severely degrades them while low-temperature *ex situ *annealing improves them. The mechanisms responsible for the degradation in the former and the improvement in the latter are discussed.

## Experiments

**Figure 1:**
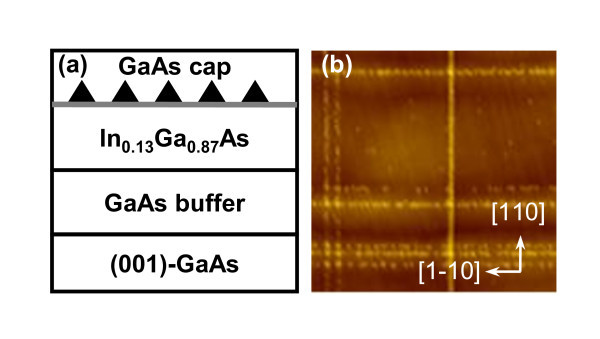
**Structure of InAs QDs on InGaAs CHPs**. (**a**) Schematic cross-sectional diagram of the QDs on CHP structure and (**b**) a 5 × 5 μm^2 ^AFM image of the uncapped QDs layer (height contrast, 11.3 nm).

The structure of InAs QDs on InGaAs CHPs under investigation is shown in the schematic cross section in Figure 1. All growth takes place in a solid-source MBE system (Riber 32P). Epi-ready (100)-GaAs substrates are prepared by standard thermal desorption of native oxides at 580°C before the deposition of 300-nm GaAs buffer layer at the same temperature followed by 50-nm In_0.13_Ga_0.87_As CHP layer at 500°C, a 30-s growth interruption, 0.80 or 0.96 monolayer (ML) of InAs at 500°C at a rate of 0.01 ML/s, another 30-s growth interruption, and the final 50-nm GaAs capping layer at 500°C. During the deposition of the InAs layer, the reflection high-energy electron diffraction spots appear, indicating the formation of QDs. The surface of the QDs grown on the CHP layer is shown in the 5 × 5 μm^2 ^atomic force microscopy (AFM) image in Figure 1b. The alignment of QDs along the orthogonal [110] and [1-10] directions occurs as a result of non-uniform surface strain fields originating from the subsurface MDs [23]. To identify the source(s) of changes in optical characteristics upon annealing, two controlled samples are grown: one is a controlled InGaAs CHP sample with identical structure to Figure 1a less the QD layer and the other is a controlled InAs QD sample (1.7-ML InAs) with identical structure to Figure 1a less the InGaAs CHP layer.

Our *in situ *annealing follows the same procedures successfully applied to conventional QDs [13,14]: the controlled QDs, the controlled CHP, and the QDs on CHP samples are removed from the growth chamber, cleaved into smaller pieces, re-attached to the molybloc, transferred back into the growth chamber, and annealed at 700°C for 10, 30, and 60 min under As_4 _partial pressure higher than 8 × 10^-6 ^Torr. Such high pressure alleviates surface As desorption, and after annealing, the surface of all samples remains reflective. For *ex situ *annealing, the samples are also cleaved into smaller pieces but later placed in the middle of a quartz tube and heated to 350°C for between 30 min and a few hours under continuous flow of a hydrogen-containing forming gas.

The optical properties of as-grown and annealed samples are characterized by macroscopic PL at 20 K. The samples are mounted on the cold finger of a closed-cycle He cryostat and excited by 476.5-nm Ar^+ ^laser at a nominal power density of *I*_0 _= 0.45 W/cm^2^. The PL signal is dispersed in a 1-m monochromator and collected by a cooled InGaAs detector using standard lock-in detection technique.

## Results and discussion

The optical properties of the as-grown and annealed QDs on CHP structure are analyzed against those of the controlled QDs and CHP samples. The results for as-grown samples will first be discussed, followed by those for samples that underwent *in situ *and *ex situ *annealing, respectively.

### As-grown

**Figure 2:**
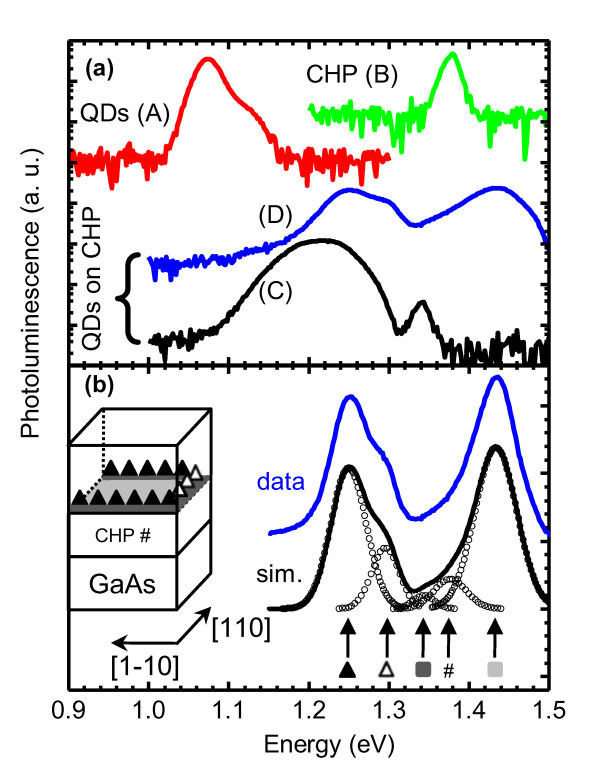
**20-K photoluminescence of the controlled QDs, the controlled CHP, and the QDs on CHP samples**. (**a**) PL spectra of the controlled QDs (sample A, in red), the controlled CHP (B, green), and the QDs on CHP samples (C, black and D, blue) at 20 K. (**b**) The measured (blue) and simulated (black) PL spectra of sample D. Open symbols (circle) are multiple Gaussian function fits. The peak energies as indicated by the arrows from left to right originate from the [1-10]-QDs, the [110]-QDs, the first WL, the CHP, and the second WL. The symbols below the arrows correspond to the structures depicted in the schematic cross-sectional diagram to the left. The vertical axis is logarithmic in (a) and linear in (b). Spectra are offset for clarity.

The 20-K photoluminescence of the controlled QDs, the controlled CHP, and the QDs on CHP samples before annealing are shown in Figure 2. The controlled QDs (sample A) show two peaks at 1.075 and 1.117 eV with corresponding full width at half maxima (FWHM) of 31 and 49 meV, respectively. The controlled CHP (sample B) shows a single emission peak at 1.377 eV with an FWHM of 21 meV. The results for QDs on CHPs are obtained from two samples destined for *in situ *(sample C) or *ex situ *(sample D) annealing. The InAs QD layer in sample C is 0.96 ML, thicker than 0.80 ML in sample D. The strong luminescence from all unannealed samples indicates that the as-grown materials are of high crystalline quality.

Sample A shows two ground states' (GSs) PL from the 1.7-ML InAs QD layer. This has been confirmed by excitation-dependent measurements. The presence of two GSs indicates that this particular growth condition on flat GaAs substrates results in QDs with a bimodal size distribution [24-26]. At higher excitation power density, two excited-state peaks resulting from the state filling of each of the GS emerge, as expected.

Sample B (CHP) is basically an InGaAs quantum well (QW) sandwiched between the GaAs buffer and GaAs capping layers. The lattice-mismatched QW is 50-nm thick, much greater than the critical thickness for strain relaxation by the formation of interfacial MDs which for In_0.13_Ga_0.87_As on GaAs is estimated at 15 nm [27]. The dislocations thus formed act as traps and non-radiative recombination centers [28]. PL from such layer is thus expected to be weak or absence. The observed peak at 1.377 eV is indeed weak with respect to sample A, yet it suggests that a significant fraction of excitons are able to combine radiatively. The 1.377-eV peak energy is higher than the bulk In_0.13_Ga_0.87_As bandgap of 1.323 eV and agrees well with the electron and hole eigenenergies estimated from self-consistent solutions of coupled Poisson-Schrodinger equation [29].

Sample C's PL exhibits a double, lopsided peak feature with the broad, low-energy lobe centered at 1.222 eV overwhelming the narrow, high-energy lobe centered at 1.344 eV. The low-energy lobe results from the 0.96-ML InAs QDs on CHP which emit at energies between those of the 1.7-ML QDs and the CHP. Its broad linewidth results from the superposition of two groups of QDs: those nucleated along the [1-10] and [110] MDs. The two peaks are not resolved in macro PL for sample C, but their presence can be deduced from the difference between the rising and falling edges, indicating the different FWHM between the [1-10]- and the [110]-aligned QDs. Different emission energies of QDs nucleated along the two orthogonal MDs have been observed in a similar structure by micro PL [9] and can be explained by direction-dependent, apparent critical thickness for QD formation [30]. The high-energy lobe centered at 1.344 eV to the right of the QDs band is attributed to the wetting layer (WL) between the InAs QDs and the underlying InGaAs CHP surface. For conventional QDs grown directly on GaAs, the low-temperature WL luminescence is centered at about 1.42 eV [31]. The 1.344 eV observed here is a result of lower confinement potential of InGaAs. Sample C is subject to *in situ *annealing, and the results are reported in the next subsection.

Sample D contains a thinner InAs layer than those in sample C. The resulting smaller QDs would thus emit at greater corresponding energies as clearly observed in Figure 2a. To elucidate the origins of all the PL peaks in sample D, with implications for C, the measured data are fitted to multiple Gaussian functions as shown in Figure 2b. The schematic cross-sectional diagram showing the layers responsible for all the PL peaks is given in the inset of the figure. The two lower PL peak energies at 1.250 and 1.296 eV are attributed to the QDs nucleated along the [1-10] and [110] MDs, respectively. The nucleation of QDs along the two orthogonal directions is asymmetrical, and previous studies have shown that QDs along the [1-10] direction are the first to form [30]; hence, their average size is larger and peak energy is smaller than that of the later-formed QDs along the orthogonal [110] direction. These two peaks are resolved in sample D but unresolved in C simply because the InAs layer in D is thinner and the QDs in both directions have not yet saturated. In sample C, the [1-10] QDs are saturated while the [110] QDs are still growing; additional In adatoms will thus get incorporated into the [1-10] QDs at a reduced rate and into the [110] QDs at an enhanced rate. Consequently, the orthogonally aligned QDs in sample C are closer in size (PL peaks less well resolved) than in D.

The next two higher-energy PL peaks at 1.344 and 1.377 eV originate from the WL and the CHP, respectively. The highest-energy PL peak at 1.42 eV is the second WL formed in the denuded zones between the cross hatches. The second WL is different from the first WL that gives rise to the 1.344-eV PL peak. The first WL is the WL between the over-critical InAs 3D *dots *and the underlying InGaAs CHP layer which exists only above the MDs. The second WL is the thin InAs 2D *film *between the InGaAs CHP layer and the overlying GaAs capping layer which exists only in the denuded zones between the cross hatches. This peak is absent in sample C because there are no denuded zones: the critical thickness for QD formation has been reached across the surface, including the areas between the cross hatches. The presence of two WLs is thus unique to sample D, but we believe that it is a general phenomenon for all Stranski-Krastanow QDs grown on cross-hatch patterns. Their existence which has not been identified until now possibly explains the more complex carrier dynamics than those exhibited in conventional QDs [32,33]. Sample D is subject to *ex situ *annealing, and the results are reported in the last subsection.

### *In situ *annealed

After 700°C *in situ *annealing, the luminescence from the QDs on CHP (sample C) is severely degraded: its PL can no longer be observed even with the shortest experimental annealing time of 10 min, in contrast to the slowly degraded PL of the controlled QDs (sample A), but similar to the controlled CHP (sample B) subject to the same annealing conditions. The degradation of the optical quality of annealed QDs on CHP thus unequivocally originates from the degradation of the CHP itself.

**Figure 3:**
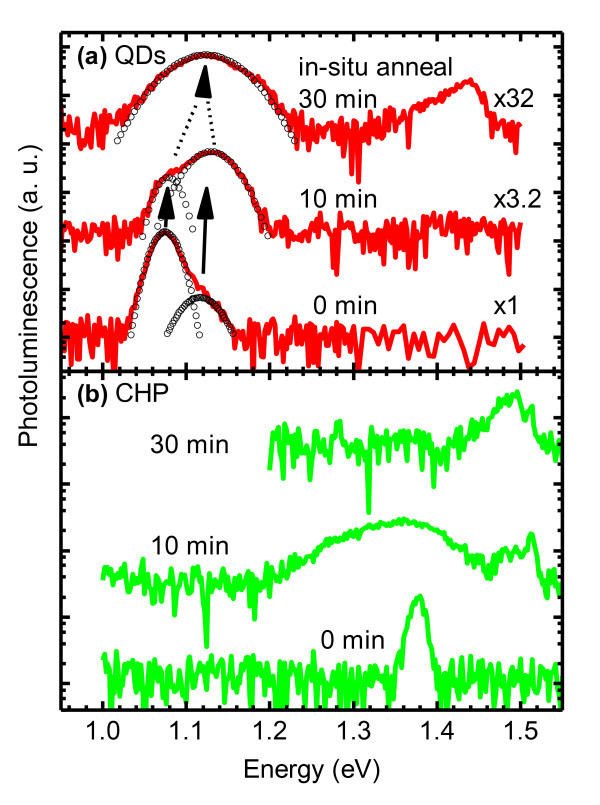
**PL spectra of samples (a) A [the controlled QDs] and (b) B [the controlled CHP]**. The samples are subject to 700°C *in situ *annealing for 0, 10, and 30 min. Spectra are offset for clarity. Symbols in (a) are multiple Gaussian function fits.

Figure 3a shows the PL of the controlled QDs after 700°C *in situ *annealing for 0, 10 and 30 min. The PL spectra of the 0- and 10-min annealed samples can be well fitted to a double Gaussian function whereas that of the 30-min annealed sample can be fitted to a single Gaussian function. The bimodal size distribution is thus maintained during the initial stages of annealing but is transformed into a monomodal one after extended annealing.

The solid arrows in Figure 3a indicate that upon 10-min annealing, the lower-energy GS peak blueshifts by 4 meV from 1.075 to 1.079 eV and the higher-energy GS peak by 13 meV from 1.117 to 1.130 eV. The observed blueshifts are much smaller than the 140 to 250 meV reported for conventional, monomodal QDs [13-19] or other nanostructures [20], yet the underlying mechanisms for the blueshifts are the same: interdiffusion and intermixing of group III cations at elevated temperatures lead to QD volume expansion, reduced confinement energy, and subsequent increased in confined electron and hole energies which have recently been modeled [34]. Prolonged annealing, however, adversely affects the optical quality of monomodal QDs [13,19] and is also the case in our bimodal QDs: the 30-min annealed sample has six times lower integrated intensity than the 10-min annealed one.

The dashed arrows in Figure 3a indicates that upon 30-min annealing, the bimodal distribution changes into a monomodal one. This is evident from two observations. First, the change in form of Gaussian fitting - from a double to a single distribution - signifies that an intermixing threshold has been reached where expanded bimodal QDs cannot be statistically distinguished. Second, the values of FWHM of the two annealed conditions are closely related. The 10-min annealed QDs with maintained bimodality exhibit two GS peaks with FWHM of 30.8 and 56.5 meV, whereas the 30-min annealed QDs exhibit one GS peak with FWHM of 87.1 meV, almost an exact linear combination of the two GS FWHM. Given finite experimental and fitting errors, the above data lead us to establish that the threshold for annealing induced transformation from bi- to monomodal QD size distribution occurs when the FWHM of the monomodal distribution equates the combined FWHM of the bimodal distribution.

Figure 3b shows the PL of the controlled CHP after 0-, 10-, and 30-min annealing. The narrow QW peak at 1.377 eV of the unannealed sample significantly broadens and is slightly red-shifted with reduced peak intensity upon 10-min annealing. Additional peak at around 1.5 eV emerges as a result of annealing. This value corresponds to exciton combination in bulk GaAs. Upon 30-min annealing, this bulk GaAs emission strengthens, whereas the QW peak weakens so much that it is below detection limits. The GaAs peak however hovers above the noise level by only a small margin, indicating a poor structural integrity.

The rapid degradation of the CHP layer and the insignificant improvement of the GaAs layers are related and not entirely unexpected. The GaAs emission comes largely from the buffer and the substrate and thus reabsorbed by the narrower gap CHP. But the CHP is compressively strained. Upon annealing, strains in zinc blende crystals with similar misfits relax via misfit and threading dislocations (TDs) [35]. MDs are confined in the growth plane, i.e., at the heterointerface, whereas TDs penetrate the layer. Improvement in GaAs layers is thus marred by the degradation of the CHP layer which explains why the deterioration of the InGaAs CHP signal is accompanied by the appearance of the weak 1.5-eV GaAs peak. For thin InGaAs sandwiched between GaAs, however, misfit strain is small and, upon annealing, interdiffusion causes a small blueshift in PL with no crystalline degradation [36]. This is not the case in our controlled CHP sample where misfit is large but necessary to induce the interfacial dislocation network that enables the formation of orthogonally aligned QDs.

The rapid degradation of the optical quality of QDs on CHP upon high-temperature *in situ *annealing thus cannot result from the degradation of QDs since the controlled QDs subject to the same annealing conditions remain optically active despite the longest annealing times as seen in Figure 3a; it must therefore result from the degradation of the CHP itself as the PL from the controlled CHP shown in Figure 3b. The thermal budget for the overlayers on QDs on CHP is thus lower than that those on conventional QDs and must be well below 700°C. Alternatively, improvement sought from post-growth annealing may be carried out *ex situ *at a lower temperature and, consequently, with a quantitatively and qualitatively different improvement. For *ex situ *annealing studies in the next section, sample D is chosen over C because of its well-resolved QD peaks and the richer PL characteristics which act as sensitive probes for material's integrity.

### *Ex situ *annealed

**Figure 4:**
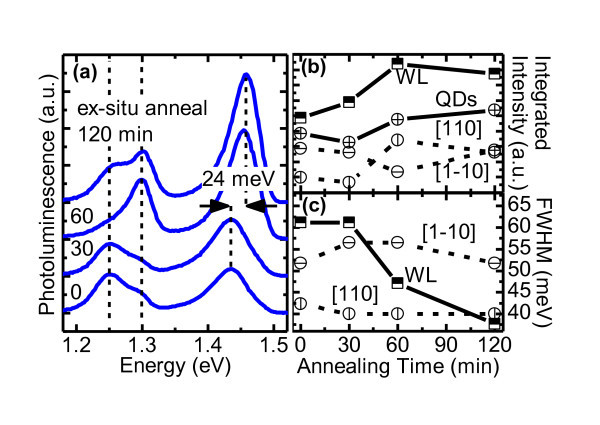
**PL spectra of sample D, and changes in integrated intensity and FWHM**. (**a**) PL spectra of sample D [QDs on CHP] subject to 350°C *ex situ *hydrogen annealing for 0, 30, 60, and 120 min (offset for clarity). Changes in (**b**) integrated intensity and (**c**) FWHM of the main emission peaks as a function of annealing time.

After 350°C *ex situ *annealing in a forming gas for between 30 and 120 min, the overall quality of the QDs on CHP (sample D) improves as shown in Figure 4a. The improvements are twofold. First, the QDs and WL emissions have overall increased intensities as shown in Figure 4b. Second, the 1.42-eV WL emission has reduced FWHM as shown in Figure 4c. This is the 1.42-eV WL, not the 1.34-eV WL whose changes upon annealing cannot be resolved as it is too close in energy to the 1.377-eV CHP peak. The improvement is not related to material crystallinity as the temperature is too low to have any effect. Instead, it is related to the abundance of hydrogen and the supplied thermal energy that is sufficiently high to dissociate the hydrogen atoms/molecules, driving them through the structure, neutralizing dislocations and dangling bonds (MDs), and making available more free carriers. Low-temperature hydrogen annealing is a standard Si process that effectively neutralizes interface-trapped charges [37] since hydrogen can diffuse several microns into Si even at room temperature [38].

Figure 4b shows the integrated intensities of the WL and the QDs as a function of annealing time. The 1.42-eV WL intensity increases immediately and significantly during the first hour of annealing after which no further improvement can be made. This emission arises from the radiative recombination of carriers photoexcited in the WL itself and those captured into the WL from the overlying GaAs capping layer. The improvement is due to the fact that the GaAs capping layer is grown at a relatively low temperature of 500°C on a lattice-mismatched layer which result in non-radiative defects and strain. In addition, the free GaAs surface is full of surface states which nullify any photoexcited surface carriers. Annealing in hydrogen makes available plentiful hydrogen atoms which subsequently permeate the epilayers and bond to dangling bonds and crystalline defects. This has two important consequences. First, it frees up carriers in the GaAs capping layer which then trickle down to the WL, increasing the intensity. Second, it relieves some strains caused by defects which induce lattice distortion. Changes in interfacial strain would result in changes in band offsets which then affect the eigenenergies of carriers confined by one or two of such interfaces. A closer inspection of Figure 4a reveals that the increased WL intensity indeed occurs together with a 24-meV blueshift, consistent with values reported by Ryu et al. who achieved similar degrees of blueshift at much higher annealing temperatures of 900°C and above [36]. Our results indicate that strains may play a much greater role than cation interdiffusion in the non-Fickian diffusion description of Ryu et al. or that cation interdiffusion readily occurs even at 350°C.

The total QD intensity in Figure 4b is obtained simply by adding the two constituent QD emissions along the [1-10] and [110] directions. Measurements by macro PL do not allow meaningful interpretation of both constituents separately since the excited beam diameter covers large areas of cross hatches and MD line densities vary across the surface. Thus, only explanation regarding the total QD emission is attempted. The total QD emission remains unchanged during the initial stages of annealing but increases slowly with annealing time after 1 h. The mechanism responsible for increased QD intensity is the same as those for increased WL intensity, only to a much smaller scale due to the comparatively lower surface coverage. The peak energies of both constituents thus remain unchanged as shown by the vertical dotted lines in Figure 4a.

Figure 4c shows the FWHM of the 1.42-eV WL peak, and the 1.250-eV [1-10]-aligned and the 1.296-eV [110]-aligned QD peaks as a function of annealing time. The changes in QDs' FWHM are non-monotonous, small, and most likely due to surface inhomogeneity, not to annealing. In contrast, the change in WL's FWHM is monotonous and large, dropping by over one third from 61 to 38 meV. The scale of change is only possible because of the relative large areal coverage of the upper (GaAs) barrier which becomes more homogeneous as more hydrogen atoms are driven to bond with random defects. The same mechanism also gives rise to the more homogeneous lower (InGaAs) layer which, being lower in energy than and adjacent to the second WL, can effectively compete for carriers and possibly explains the decrease in WL intensity and the increase in QD intensity after 120-min annealing seen in Figure 4b. The decrease in WL intensity is due to carrier transfer to the more energetically favorable InGaAs CHP. The increase in QD intensity is due to the InGaAs CHP channeling some of these new carriers through the 1.344-eV WL where they are subsequently captured by the QDs. Low-temperature *ex situ *annealing thus proves to be a viable approach for enhancing optical emissions from InAs QDs on InGaAs CHPs while maintaining the rich optical feature.

## Conclusions

InAs QDs on InGaAs CHPs are grown by MBE, characterized by low-temperature PL, and found to be optically active in the 1.1 to 1.4 eV range with distinct emission peaks from the orthogonally aligned [1-10] and [110] InAs QDs, two different wetting layers, and the InGaAs CHP. The PL spectra of the QDs on CHPs are quenched when the structure is subject to 700°C *in situ *annealing. In separate controlled experiments, QDs are found to survive the same treatments whereas the CHP deteriorated. The quenching thus results from CHP deterioration, most likely driven by strain relaxation via the formation of additional misfit and threading dislocations which are effective carrier traps. When subject to 350°C *ex situ *hydrogen annealing, however, the structure shows an increase in overall PL intensity, a small blueshift accompanied by spectral narrowing for the 1.42-eV WL. Hydrogen bonding is believed to cause such improvement as it is effective at neutralizing defects and relieving associated strains which frees up carriers and smoothens band discontinuities along heterointerfaces.

## Abbreviations

AFM: atomic force microscopy; CHP: cross-hatch pattern; FWHM: full width at half maximum; GS: ground state; MBE: molecular beam epitaxy; MD: misfit dislocation; ML: monolayer; PL: photoluminescence; QD: quantum dot; QW: quantum well; TD: threading dislocation; WL: wetting layer.

## Competing interests

The authors declare that they have no competing interests.

## Authors' contributions

CH grew and measured the MBE samples, and interpreted the PL spectra. SP provided helps, obtained funding, and supervised the group. SK conceived, designed, and supervised the experiments; obtained funding; analyzed the data; and wrote the manuscript.
